# Mixed infected laryngocoele presenting as airway obstruction: a case report

**DOI:** 10.1093/jscr/rjaa615

**Published:** 2021-02-28

**Authors:** Danielle L James, Stephen Garry, Mel Corbett, John Lang

**Affiliations:** Department of Otorhinolaryngology, University Hospital Galway, Galway, Ireland; Department of Otorhinolaryngology, University Hospital Galway, Galway, Ireland; Department of Otorhinolaryngology, University Hospital Galway, Galway, Ireland; Department of Otorhinolaryngology, University Hospital Galway, Galway, Ireland

## Abstract

Laryngocoele is a rare entity, defined as an abnormal cystic dilatation of saccule of the laryngeal ventricle. Three types of laryngocele have been described, based on their relation to the thyrohyoid membrane: internal, external or mixed type. Symptoms are variable, including neck swelling, shortness of breath, dysphonia and fever, if the laryngocoele becomes infected. Patients may also present in extremis with airway obstruction. We present the case of a healthy 34-year-old gentleman with acute airway obstruction due to a mixed infected laryngocoele. Flexible nasoendoscopy showed a large cystic swelling arising from the laryngeal ventricle. Computed tomography of neck confirmed a right paraglottic collection extending into the ventricle and glottis, causing significant airway compromise. The patient was managed with microlaryngoscopy and cystic decompression. At outpatient follow up, he was completely asymptomatic and is currently under surveillance. Endoscopic decompression is a safe and effective initial management for mixed laryngocoele.

## INTRODUCTION

Laryngocoele is a rare entity with an incidence of 1 in 2.5 million [1]. It forms as a result of air-filled dilation of the saccule of the laryngeal ventricle, communicating with the laryngeal lumen. It may be congenital in nature or acquired due to increased intralaryngeal pressure. Laryngopyocoele is a rare complication, occurring in 8% of patients [1, 2]. The neck of the ventricle becomes obstructed (e.g. tumour, inflammation), and the mucus secreted by the lining of the saccule collects within the laryngocoele, becoming infected. This may cause acute airway obstruction, warranting emergency management. The symptom patients’ present with is based on the anatomical location of the laryngocoele. Three types of laryngocele have been described, based on their relation to the thyrohyoid membrane: internal, where the sac remains within the confines of the thyroid cartilage, external, where the sac may protrude through the thyrohyoid membrane and present as an anterior neck mass, or mixedtype.

We report an unusual case of an otherwise fit and healthy 34-year-old male who presented in airway extremis due to a mixed laryngopyocoele. His symptoms were managed successfully with endoscopic decompression and marsupialization. We present this as an alternative operative approach to standard external approach favoured in the literature.

**Figure 1:**
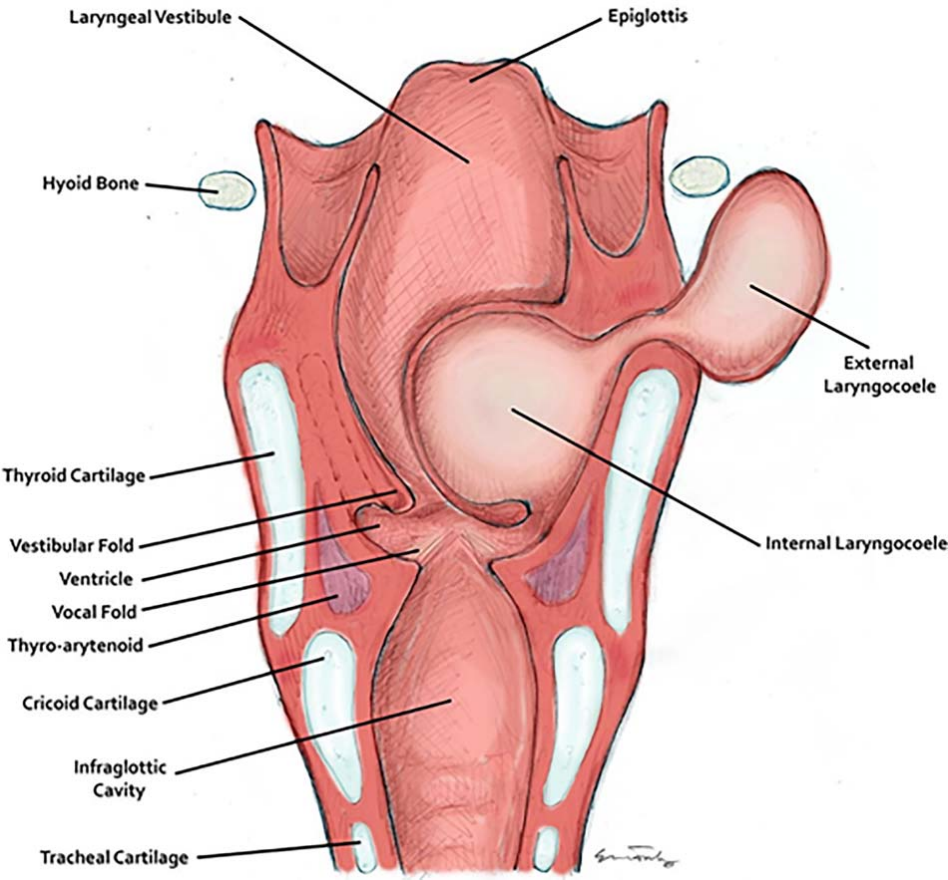
The anatomy of the larynx and development of a laryngocoele. This figure was drawn and provided by Mr. Mel Corbett.

**Figure 2:**
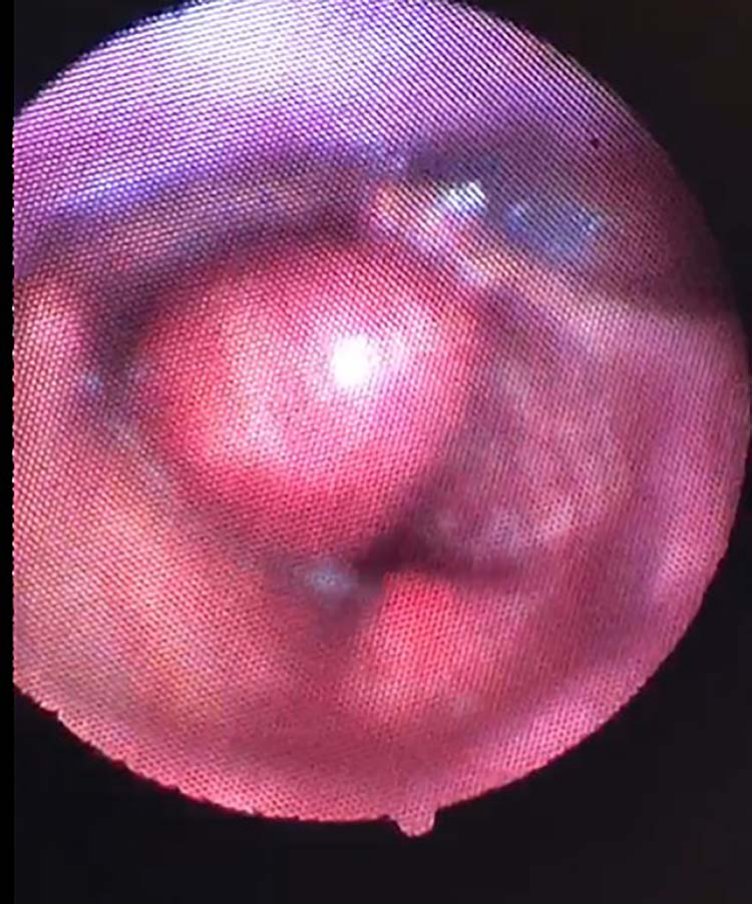
Clinical photograph showing the large cystic swelling arising from the right ventricle.

**Figure 3:**
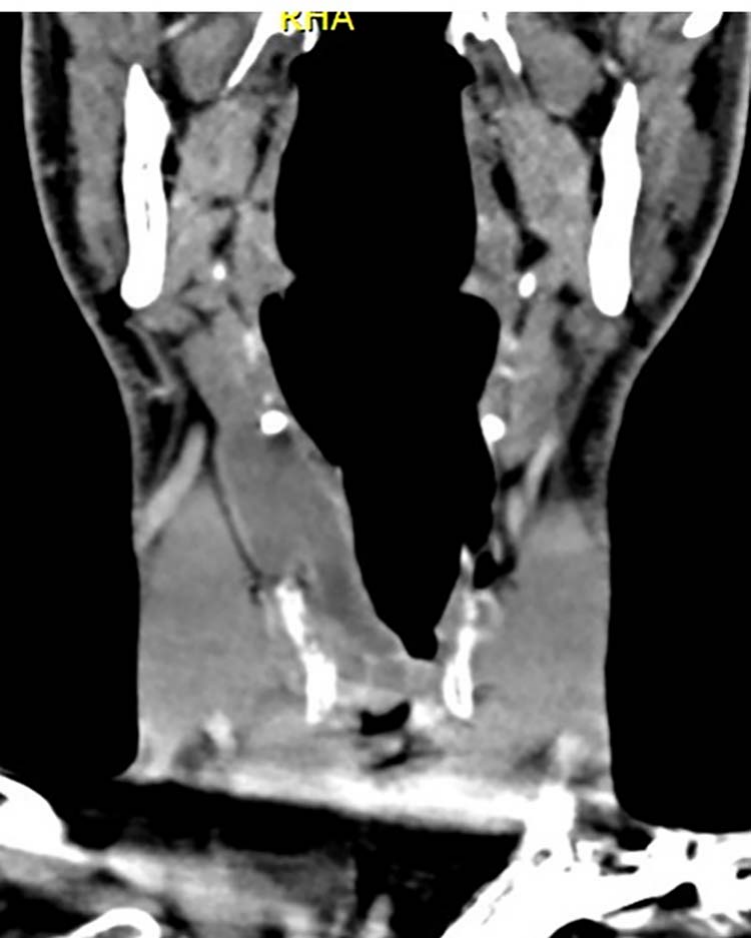
Coronal CT of neck showing the internal and external components of the laryngocoele, in relation to the thyrohyoid membrane.

**Figure 4:**
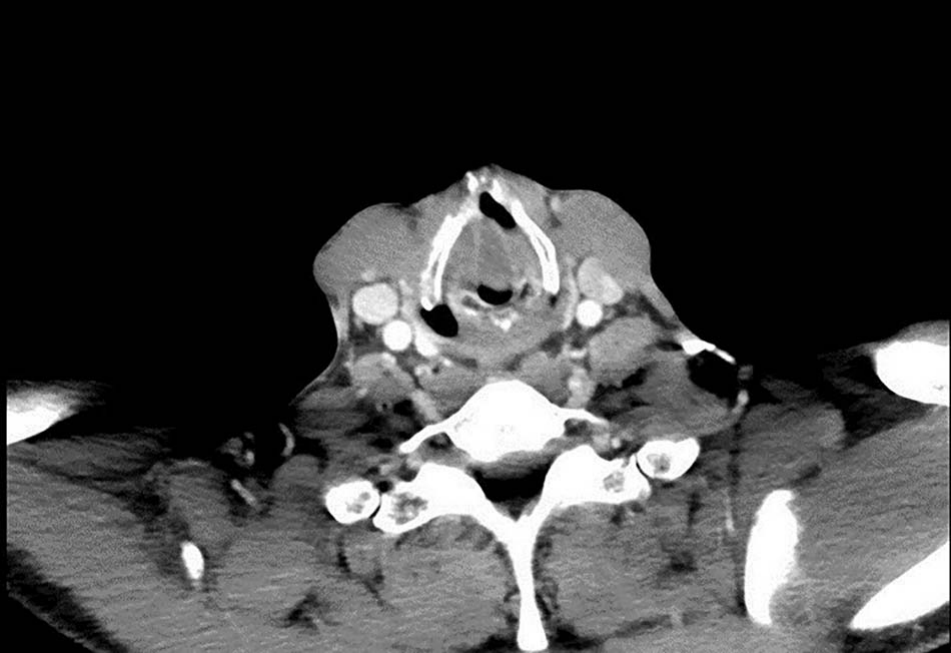
Axial CT showing obstruction at the level of the vocal cords. Media: video showing the laryngocoele.

## CASE REPORT

Our patient presented with a 2-day history of stridor and dyspnoea with a preceding 2-month history of hoarseness and progressive positional dyspnoea. One week prior to his presentation, due to progression of symptoms, he sought medical attention and was commenced on antibiotics for a presumed bacterial pharyngitis; however, his condition continued to worsen prompting his presentation to the emergency department. His past medical history was insignificant, but notably he was a recent ex-smoker and works in the agriculture sector, thus was heavy lifting and straining on a daily basis.

On examination, he was tachypnoeic, with a respiratory rate of 32 and oxygen saturations of 92% on room air. Although he was afebrile and haemodynamically stable, he was visibly exhausted, with accessory muscle use and stridulous breathing. There were no palpable swellings in the patient’s neck. Flexible nasoendoscopy showed a large submucosal swelling arising from the right vestibular fold, causing intermittent airway obstruction (Video) (Fig. 2). Laboratory markers were within normal range. Computed tomography of neck showed a well-defined, peripherally enhancing collection in right paraglottic space, extending from level of hyoid bone into the right laryngeal ventricle, with significant airway compromise at the level of the vocal cords (Figs 3 and 4).

The patient was admitted and started on intravenous antibiotics, intravenous steroids and nebulized adrenaline in an effort to improve his airway symptoms. Initially, he responded well; however, he still had an increased work of breathing and began totire.

Upon discussion with the consultant Otolaryngologist and consultant Anaesthetist, the consensus was this was most likely an infected laryngocoele and would be amenable to endoscopic decompression. The patient was brought to theatre expediently and the airway was secured by an awake fibre optic intubation. Suspension laryngoscopy was performed and the laryngocoele was de-roofed and de-compressed using long laryngeal biopsy forceps. The laryngocoele contained purulent, mucoid fluid, which was aspirated, and a sample was taken for culture and sensitivity. The excess mucosa was excised and sent to histology and marsupialization of the mucosal edges performed. The patient was extubated without difficulty and was discharged the followingday.

At his 6-month outpatient follow up, the patient was completely symptom free. His voice had returned to normal and flexible nasoendoscopy revealed a patent airway and no sign of recurrence of the laryngocoele. He continues to be followed up closely.

## DISCUSSION

Laryngocele is an air- or fluid-filled herniation of the laryngeal saccule [1]. The saccule is a blind-ending structure that opens into the laryngeal ventricle, the lateral space between the true vocal cords and vestibular folds, as seen in Fig. 1.

Laryngocoeles may be congenital or acquired. Congenital laryngocele develops due to the presence of a congenital saccular dilatation of the ventricular appendage. Increased intralaryngeal pressure may be brought about by straining activities (e.g. heavy lifting), which fix the diaphragm in forced expiration against a closed larynx or modified valsalva manoeuvres, e.g. wind instrument playing, resulting in increased glottic pressure [3]. Furthermore, there is the well-documented association of laryngocoele with laryngeal carcinoma [4]. The presence of a supraglottic carcinoma may result in a valve like closure at the neck of the ventricular appendage, allowing the entrance of air but preventing its exit [5]; hence, patients with laryngocoeles should be worked up accordingly.

The clinical presentation is variable, dictated in part by the anatomical location. Internal laryngocele typically presents with hoarseness, dyspnoea and a foreign body sensation. External laryngocele may present as a smooth, compressible mass in the lateral aspect of the neck. Mixed laryngocele may present with symptoms of both internal and external components, although interestingly our patient did not have signs of any external component.

Our patient required emergency surgery due to his threatened airway. As laryngoceles are not commonly encountered entities, there is little consensus regarding their management. Zelenik *et al*. [6] recently conducted a meta-analysis of the surgical management of laryngocoeles, comprising 41 case reports and nine case series, encompassing a total of 86 patients. Although external approach via lateral thyrotomy is currently preferred for external or combined laryngocoeles, endoscopic resection is favoured for internal noninfected laryngocele [6, 7]. Internal laryngopyocoeles are scarcely reported in the literature, often proving difficult to manage [8]. Securing a definitive airway is of paramount importance, and often a tracheostomy is required; however, in our case, awake fibre optic intubation with subsequent decompression proved successful. The first case of internal laryngopyocoele managed successfully by endoscopic decompression was reported in 2011, with few reported thereafter [9]. Equally, our case appears to have been managed successfully by endoscopic marsupialization; thus, the authors advocate for this approach in the acute setting. He will require close surveillance to monitor for recurrence of his condition, at which point he may go on to require a formal resection using an external approach.

## CONFLICT OF INTEREST STATEMENT

None declared.

## FUNDING

None.

## Supplementary Material

Media1_rjaa615
